# ^68^Ga-DOTATOC PET/CT-Based Radiomic Analysis and PRRT Outcome: A Preliminary Evaluation Based on an Exploratory Radiomic Analysis on Two Patients

**DOI:** 10.3389/fmed.2020.601853

**Published:** 2021-01-26

**Authors:** Virginia Liberini, Osvaldo Rampado, Elena Gallio, Bruno De Santi, Francesco Ceci, Beatrice Dionisi, Philippe Thuillier, Libero Ciuffreda, Alessandro Piovesan, Federica Fioroni, Annibale Versari, Filippo Molinari, Désirée Deandreis

**Affiliations:** ^1^Nuclear Medicine Unit, Department of Medical Sciences, University of Turin, Turin, Italy; ^2^Medical Physics Unit, Azienda Ospedaliero-Universitaria Città della Salute e della Scienza, Turin, Italy; ^3^Biolab, Department of Electronics and Telecommunications, Politecnico di Torino, Turin, Italy; ^4^Department of Endocrinology, University Hospital of Brest, Brest, France; ^5^Medical Oncology Division 1, Azienda Ospedaliero-Universitaria Città della Salute e della Scienza, University of Turin, Turin, Italy; ^6^Division of Oncological Endocrinology, Department of Medical Sciences, University of Turin, Turin, Italy; ^7^Medical Physics Unit, Azienda Unit Sanitaria Locale di Reggio Emilia - Istituto di Ricovero e Cura a Carattere Scientifico of Reggio Emilia, Reggio Emilia, Italy; ^8^Nuclear Medicine Unit, Azienda Unit Sanitaria Locale di Reggio Emilia - Istituto di Ricovero e Cura a Carattere Scientifico of Reggio Emilia, Reggio Emilia, Italy

**Keywords:** ^68^Ga-DOTATOC PET/CT, peptide receptor radionuclide therapy, radiomic analysis, NET, total lesion somatostatin receptor expression, somatostatin receptor expressing tumor volume

## Abstract

**Aim:** This work aims to evaluate whether the radiomic features extracted by 68Ga-DOTATOC-PET/CT of two patients are associated with the response to peptide receptor radionuclide therapy (PRRT) in patients affected by neuroendocrine tumor (NET).

**Methods:** This is a pilot report in two NET patients who experienced a discordant response to PRRT (responder vs. non-responder) according to RECIST1.1. The patients presented with liver metastasis from the rectum and pancreas G3-NET, respectively. Whole-body total-lesion somatostatin receptor-expression (TLSREwb-50) and somatostatin receptor-expressing tumor volume (SRETV wb-50) were obtained in pre- and post-PRRT PET/CT. Radiomic analysis was performed, extracting 38 radiomic features (RFs) from the patients' lesions. The Mann–Whitney test was used to compare RFs in the responder patient vs. the non-responder patient. Pearson correlation and principal component analysis (PCA) were used to evaluate the correlation and independence of the different RFs.

**Results:** TLSREwb-50 and SRETVwb-50 modifications correlate with RECIST1.1 response. A total of 28 RFs extracted on pre-therapy PET/CT showed significant differences between the two patients in the Mann–Whitney test (*p* < 0.05). A total of seven second-order features, with poor correlation with SUVmax and PET volume, were identified by the Pearson correlation matrix. Finally, the first two PCA principal components explain 83.8% of total variance.

**Conclusion:** TLSREwb-50 and SRETVwb-50 are parameters that might be used to predict and to assess the PET response to PRRT. RFs might have a role in defining inter-patient heterogeneity and in the prediction of therapy response. It is important to implement future studies with larger and more homogeneous patient populations to confirm the efficacy of these biomarkers.

## Introduction

Peptide receptor radionuclide therapy (PRRT) has proven to be an effective treatment for metastatic gastroenteropancreatic (GEP) neuroendocrine tumors (NET) (1). ^177^Lu-DOTATATE has been approved by the European Medicine Agency in 2017 for treating inoperable or metastatic GEP-NET with progressive disease. To evaluate the response to PRRT, the Delphic consensus assessment for GEP-NET (2) considers suboptimal both the Response Evaluation Criteria in Solid Tumors 1.1 (RECIST 1.1) and positron emission tomography (PET) parameters derived by functional imaging (standardize uptake value, SUV), considering the high variability in somatostatin receptor expression and the different histological patterns related to disease heterogeneity. PET/computed tomography (CT) allows one to evaluate the *in vivo* expression of the somatostatin receptor (SSTR) in NET (^68^Ga-DOTA-SSTR PET) and is considered a gatekeeper to select the proper candidate to PRRT (3–5). Thus, the identification of new and reliable semi-quantitative and quantitative imaging parameters (e.g., using radiomic analysis) might be crucial to better select eligible patients and to assess the response to PRRT. Radiomic is a new innovative bioinformatic approach to the image's analysis. Through the use of standardized mathematical-based models, radiomic allows one to evaluate tumor heterogeneity and quantify predictive and prognostic parameters, radiomic features (RFs), that can be applied in clinical decision support system and in clinical research (6–8).

We hypothesize that advanced semi-quantitative PET parameters and radiomic analysis applied to ^68^Ga-DOTA-TOC PET/CT might correctly identify tumoral heterogeneity and new parameters able to predict response to PRRT in NET patients. In this preliminary study, we retrospectively explored this hypothesis on two NET patients with liver metastases and different outcome from PRRT therapy.

## Materials and Methods

### Patient Population

We retrospectively performed semiquantitative and radiomic analysis in the ^68^Ga-DOTATOC PET/CT image of two patients both presenting with NET liver metastases, selected from a retrospective study approved by the Local Ethical Committee (IRB protocol: CS2/477) of AOU Città della Salute e della Scienza. Both patients were treated with PRRT in a clinical trial (EUDRACT 2015-005546-63) approved by the Ethical Committee of “Area Vasta Emilia Nord” (AVEN) of the “Azienda USL-IRCCS of Reggio Emilia, Italy.”

Patient A had metastatic disease by G3 NET of rectum and patient B by G3 NET of the pancreas. Both patients underwent surgery as primary therapy (pT2N0, Ki67 10%) plus somatostatin analog (lanreotide) administration. Both patients developed liver metastases and treated with multiple radiofrequency ablations, subsequently with chemotherapy after progression, and later with everolimus. Liver biopsy revealed similar Ki67% patterns (Ki67 25% for patient A and 22% for patient B). PRRT was considered as the third line of treatment according to clinical trial inclusion criteria. ^18^FDG PET/CT showed a faint uptake in the lesions, with NET-PET score of 2a for patient A and score of 1 for patient B (4), while the ^68^Ga-DOTATOC PET/CT scan showed a visually high uptake in all lesions. Six cycles of PRRT were administered, completed in November 2018 for patient A and in July 2018 for patient B.

Patient A and patient B, according to the trial design, underwent several ^177^Lu- and ^90^Y-DOTATATOC administrations. Dosimetry was conducted at the first cycle of therapy after a therapeutic injection of ^177^Lu-DOTATATOC, assuming that a complete dosimetric evaluation at the first cycle is a close approximation of the absorbed dose on subsequent treatments (9, 10). The clinical trial also included a SPECT/CT acquisition performed 24 h after therapy at cycles 3 and 6, which excluded a significant variation in single tumor volumes considered for dosimetric purposes.

Tumor-absorbed doses were calculated following the procedure described by Finocchiaro and Murray (11). Similar biodistribution and kinetics for peptides labeled with ^177^Lu and ^90^Y were generally assumed (12); therefore, the results obtained with ^177^Lu were extrapolated to ^90^Y, simply substituting physical decay constant λ and *S* factor, as reported in the paper of Guerriero et al. (13).

**Figure 1:**
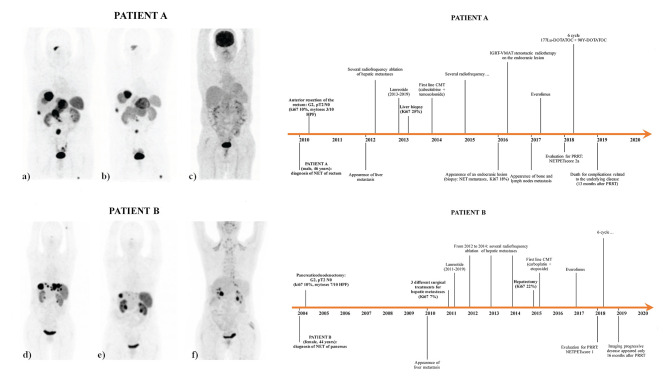
^68^Ga-DOTATOC PET/CT [pre-therapy **(a)** and post-therapy **(b)**] and ^18^FDG PET/CT [pre-therapy **(c)**] of patient A, ^68^Ga-DOTATOC PET/CT [pre-therapy **(d)** and post-therapy **(e)**] and ^18^FDG PET/CT [pre-therapy **(f)**] of patient B, and patient history timeline of both patient A and patient B. The pre- and post-therapy maximum intensity projection (MIP) of the ^68^Ga-DOTATOC PET/CT of patient B, respectively, shows a homogeneous decrease in size and somatostatin receptor expression of all the liver lesions. Patient A received one cycle of ^90^Y-DOTATOC (1.7 GBq) followed by six cycles of ^177^Lu DOTATOC (total cumulated activity, 26.13 GBq), with a median estimated absorbed dose of 104.5 Gy (range: 63–134). Patient B received three consecutive cycles of ^90^Y-DOTATOC (cumulated activity of 3 GBq) followed by three cycles of ^177^Lu DOTATOC (cumulated activity of 14.30 GBq), with a median estimated absorbed dose of 104 Gy (range: 94–122).

Patient A received only one cycle of ^90^Y due to radioisotope supply problems. However, the range of tumor-absorbed doses for patient A (63–134 Gy) was comparable to the tumor-absorbed doses for patient B (91–122 Gy). Patient A died for cancer-related disease 13 months after treatment, while patient B at the end of follow-up was alive with persistent disease and presented disease progression 16 months after PRRT (overall survival was 26 months after PRRT). Patients' information and the dosimetry schedule for both patients are summarized in Figure 1.

### Image Acquisition

The patients underwent ^68^Ga-DOTATOC PET/CT before and after the end of PRRT treatment. According to RECIST 1.1, patient A was considered as non-responder to PRRT, while patient B achieved very good partial response. All PET/CT scans were performed on the same scanner (Philips Gemini Dual-slice EXP, Philips Medical Systems, Cleveland, OH, USA) at the University Hospital of Turin. Radiopharmaceutical synthesis and PET/CT images were performed as recommended by international guidelines (3, 14, 15).

### Quantitative Imaging Analysis

Radiomic analysis was performed by manually contouring the VOI by one operator (VL) using the software LIFEx v.5.10 (IMIV/CEA, Orsay, France) (16). An absolute intensity rescaling factor of 0–60 of the SUV of the VOI was applied (64 bins, 0.95 fixed bin width). A total of 38 features were extracted: six conventional PET parameters, six descriptors of the image intensity histogram (skewness, kurtosis, excess kurtosis, energy, entropy-log2, and -log10), four shape-based features, 22 second-order statistics texture signatures from all VOI >64 voxels (gray-level co-occurrence matrix, GLCM; gray-level run length matrix, GLRLM; gray-level zone length matrix, GLZLM; and neighborhood gray-level different matrix, NGLDM). The compliance of Lifex feature calculation formulas with the IBSI standard was verified (17). Furthermore, the estimated absorbed dose in target lesions with higher uptake was evaluated as previously described (18).

In both pre- and post-PRRT PET scans, two volumetric parameters (19) were also evaluated: the somatostatin receptor expressing tumor volume (SRETV), representing the volume of the isocontouring-derived volumes of interest (VOI) based on percentage of 50% threshold of lesion maximum SUV (VOI_50_) (20), and the total lesion somatostatin receptor expression (TLSRE), calculated by multiplying the SRETV of each lesion with its corresponding SUV mean value. Moreover, the whole-body SRETV (SRETV_wb−50_) and TLSRE (TLSRE_wb−50_) of each patient were also calculated in both pre- and after-PRRT scan.

### Statistical Analyses

Mann–Whitney test was used to compare the RFs extracted by the VOI of the liver metastasis lesion of the two patients on the pre-PRRT scan. To evaluate the independence of the features, the correlation of each RF with all the others was studied using regression analysis, generating a Pearson correlation matrix. Two RFs were considered strongly correlated in case of a correlation coefficient >0.8 or lower than −0.8. Only RFs which were not strongly correlated with SUVmax and lesion PET volume were analyzed due to their already established role on PRRT as predictive and prognostic biomarkers (21–26). Finally, principal component analysis (PCA) was used to obtain an alternative visualization of correlated and independent RFs and to investigate the possibility of creating a smaller set of maximally uncorrelated RFs (principal components) able to explain the majority of total variation in the data set. All statistical analyses were performed using R software (www.rstudio.com).

## Results

The response to therapy was more heterogenous in patient A, with some liver and abdominal lesions increased in size and others showing a partial response. A total of eight liver metastases in patient A and 10 liver metastases in patient B were considered for inter-patient RF comparison (Supplementary Table 1). Moreover, in patient A, two lymph nodes and two bone lesions were further analyzed.

**Figure 2:**
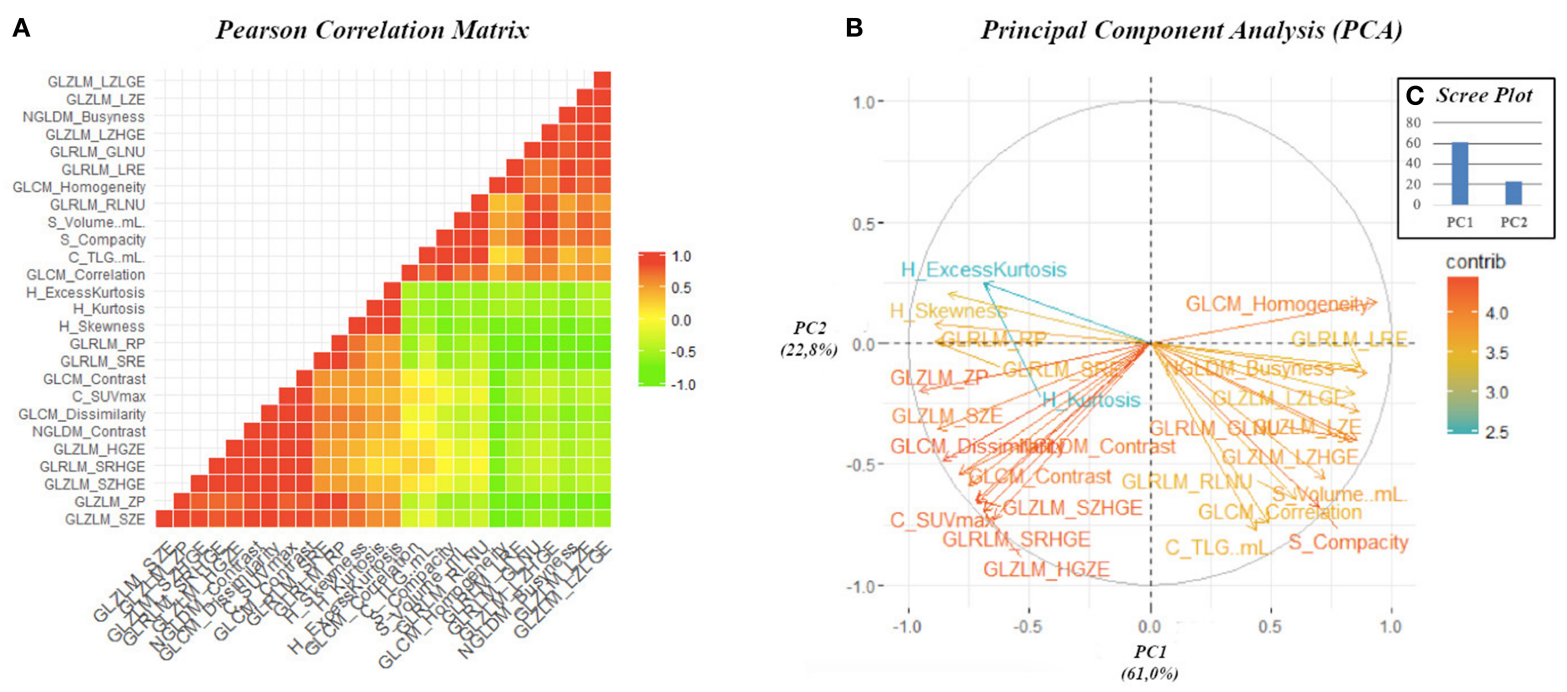
Pearson correlation matrix heat map, considering only the 26 radiomic features, resulted significant in the Mann–Whitney test **(A)**. This graphic displays the absolute value of the correlation coefficient between each pair of radiomic features, ranging from + 1 (positive linear correlation, in red) to −1 (negative linear correlation, in green). The correlation coefficient of 0 is represented in yellow and identified radiomic features that are not correlated. Graphic representation of the feature correlation plots resulting from the principal component analysis performed on the 26 radiomic features resulted significant in the Mann–Whitney test **(B)**. It shows the relationships between features: positively correlated features are grouped together, and negatively correlated features are positioned on the opposite quadrants. The distance between features and the origin measures the quality of the features on the factor map, and features that are away from the origin are well represented on the factor map. The standardize uptake value-related radiomic features (RFs) are visible in the lower left quadrant and the volume-related RFs in the lower right. The scree plot of the percentages of variation that each principal component account for shows that PC1 and PC2 identified 83.8% of the percentages of variation **(C)**.

**Figure 3:**
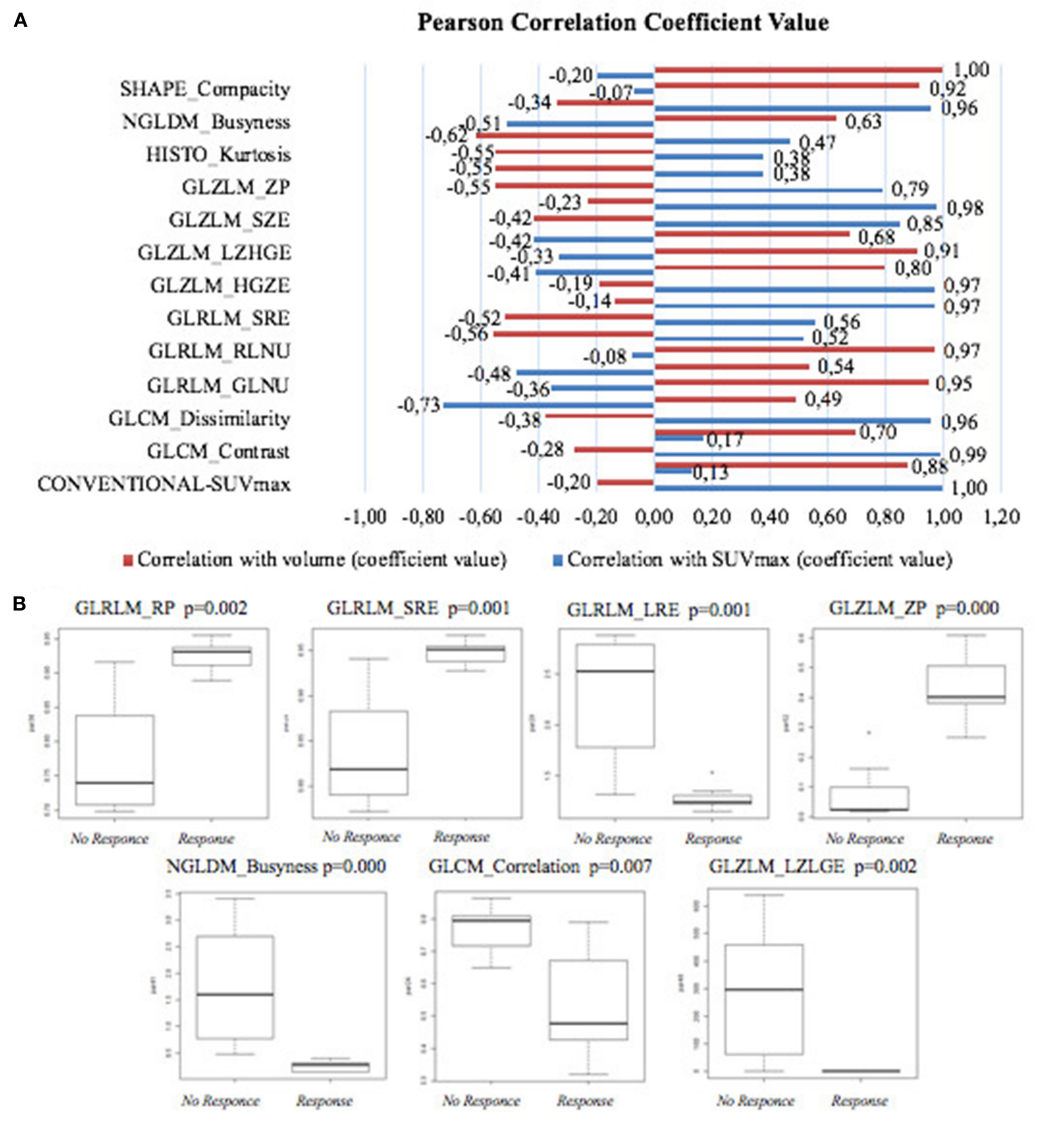
**(A)** Pearson correlation coefficients between radiomic features and SUVmax and volume (ml), respectively. **(B)** Boxplot of the second-order features not correlated with both SUVmax and volume in non-responder and responder.

Comparing the liver metastases of pre-PRRT scan, 28 RFs resulted significantly different between patients A and B in the Mann–Whitney test (Supplementary Table 2). Figure 2A shows the results of the Pearson correlation to identify the non-redundant features. The relative correlation of these RFs with SUVmax and volume are shown in Figure 3A. Seven second-order features resulted as not correlated with both SUVmax and volume and statistically different between patients A and B (Figure 3B). Table 1 summarizes the dosimetry data, SUVmax, PET volume, and seven RF values' variation in pre- and post-PRRT scan in the liver (patients A and B), lymph nodes, and bone lesions (patient A). Finally, the PCA of the first two PCs, performed in 26 features, explained 83.8% of total variance (Figures 2B,C).

**Table 1:** Dosimetry data extracted by the first cycle of therapy.

**Lesions**	**Dosimetry data**		^****68****^**Ga-DOTATOC PET/CT data**										
	**Volume (cc)**	**Total dose (Gy)**	**PET/CT**	**SUVmax**	**Volume (ml)**	**Volume (voxel)**	**GLCM correlation**	**GLRLM SRE**	**GLRLM LRE**	**GLRLM RP**	**NGLDM-Busyness**	**GLZLM LZLGE**	**GLZLM ZP**
**PATIENT A**													
Liver 1	292.3	63	Pre	17.72	450.69	7,042	0.86	0.76	3.35	0.67	4.32	2,643.28	0.01
			Post	17.81	868.61	13,572	0.86	0.77	3.22	0.68	8.51	6,788.67	0.01
Liver 7	89.8	134	Pre	35.89	182.02	2,844	0.79	0.92	1.44	0.89	0.89	6.3	0.19
			Post	27.06	139.78	2,184	0.77	0.91	1.56	0.87	0.84	6.13	0.12
Liver 6	4.1	113	Pre	14.1	18.69	292	0.65	0.89	1.58	0.86	0.98	74.64	0.06
			Post	17.07	23.42	366	0.64	0.91	1.5	0.87	1.04	37.32	0.11
Lymph node 1	40.3	108	Pre	19.15	134.91	2,108	0.76	0.87	1.91	0.81	2.34	185.47	0.04
			Post	9.45	377.41	5,897	0.79	0.68	4.69	0.58	11.55	9,010.93	0.01
Lymph node 2	6.4	199	Pre	25.16	41.47	648	0.73	0.93	1.43	0.89	1.12	26.8	0.18
			Post	21.09	17.22	269	0.61	0.92	1.46	0.89	0.72	19.82	0.2
Bone 1	6.3	11	Pre	11.84	36.22	566	0.63	0.8	2.35	0.74	1.89	351.86	0.05
			Post	12.16	72.9	1,139	0.68	0.77	2.72	0.71	2.64	1,006.04	0.02
Bone 2	7	80	Pre	19.22	19.14	299	0.62	0.91	1.48	0.88	0.76	29.32	0.17
			Post	11.01	34.3	536	0.64	0.87	1.8	0.83	3.35	385.08	0.03
**PATIENT B**													
Liver 1	17.6	115	Pre	93.14	81.54	1274	0.80	0.96	1.23	0.94	0.24	5.57	0.49
			Post	63.73	30.72	480	0.70	0.93	1.42	0.9	0.38	17.32	0.3
Liver 6	12.6	97	Pre	104.4	44.22	691	0.68	0.96	1.21	0.94	0.21	1.18	0.51
			Post	3.92	4,93	77	0.27	0.7	2.88	0.68	1.98	185.25	0.05
Liver 7	4.15	94	Pre	85.51	24.64	385	0.65	0.95	1.26	0.93	0.21	3.68	0.42
			Post	5.01	4.74	74	0.35	0.77	2.46	0.73	1.4	148.17	0.05
Liver 9	6.4	122	Pre	76.17	37.76	590	0.68	0.97	1.16	0.95	0.22	0.46	0.53
			Post	6.88	7.1	111	0.40	0.81	2.16	0.77	1.55	197.32	0.05

*Values of the SUVmax, PET volume, and the seven radiomic features of second order not associated with both SUVmax and volume (GLCM-Correlation, GLRLM-LRE, GLRLM-RP, GLRLM-SRE, GLZLM-LZLGE, GLZLM-ZP, and NGLDM-Busyness) extracted by the pre- and post-PRRT PET/CT scan*.

Regarding the two volumetric parameters, baseline TLSRE_wb−50_ and SRETV_wb−50_ were 5,524.7 and 493.8 ml for patient A and 1,780.2 and 35.2 ml for patient B, respectively. At the post-PRRT PET scan, both TLSRE _wb−50_ and SRETV_wb−50_ increased in patient A [TLSRE_wb−50_ 9,291.6 ml (ΔTLSRE_wb−50_ +68.2%) and SRETV_wb−50_ 991.0 ml (ΔSRETV_wb−50_ +100.7%)] and decreased in patient B [TLSRE_wb−50_ 202.4 ml (ΔTLSRE_wb−50_ −88.6%) and SRETV_wb−50_ 23.2 ml (ΔSRETV_wb−50_ −51.7%)].

## Discussion

There is a lack of validated quantitative parameters able to predict the response to PRRT in PET imaging, while radiomic approach is emerging as a very promising analysis to study tumoral heterogeneity and should be evaluated for its prognostic and predictive role. Werner et al. (27) analyzed RFs on ^68^Ga-DOTA peptides in 31 patients with G1/G2 pancreatic NET. They found that “TF entropy” (corresponding to GLCLM entropy) was associated with overall survival (cutoff = 6.7, *p* = 0.02), and increasing entropy might be a predictor of longer survival. In our study, the median value of GLCLM entropy was >6.7 for patient B and <6.7 for patient A, even if not reaching statistical significance. The Mann–Whitney test demonstrated a significant difference between the two patients in 28 other RFs on baseline PET/CT, which could be related to differences in lesion behavior. Seven second-order RFs have been identified as poorly associated with SUVmax and PET volume parameters and might be considered as potential predictors of therapy response.

In the post-PRRT PET/CT scan of patient B, the value of GLRLM-LRE and NGLDM-Busyness increased in liver responder lesions (“liver 6, 7, and 8” with a decrease of SUVmax and PET volume). On the contrary, GLCM correlation, GLRLM-SRE, GLRLM-RP, and GLZLM-ZP decreased in the same lesions (data are shown in Table 1). Furthermore, in a lesion (“lymph node 1”) of patient A, characterized by decreasing SUVmax, these RFs showed similar changes with the only exception of GLCM correlation, despite the increasing PET volume (stable disease for RECIST 1.1). These changes have not been observed in non-responder lesions in both patients; in particular, GLRLM SRE, GLRLM LRE, GLRLM RP, GLZLM ZP, and NGLDM-Busyness did not change consistently, resulting to be almost stable. Finally, GLZLM-LZLGE changes seem less related to the PRRT response, as increasing and decreasing changes have been observed in both responder and non-responder lesions randomly. Furthermore, RF changes seem to be independent from the Gy delivered to the lesions.

These data suggest the possibility to assess the response to PRRT through the evaluation of the changes of these RFs in post-therapy PET/CT scan if confirmed trough a prospective study (2).

Finally, PCA was performed to reduce data dimensionality and to build two new independent variables from the radiomic features which were able to describe 83.8% of total variance. This result suggests that PCA might be useful in reducing the complexity of the radiomic model, avoiding redundant data, but maintaining relevant information about lesion characteristics in PET image to predict the response to PRRT. While far from being definitive, these data allow one to hypothesize a potential role both for RFs in pre-therapy scan and ΔRF changes as predictor of therapy response, in combination with predictive parameters (including standard semiquantitative PET parameters and dosimetry) (2, 4, 8, 19).

Recently, Weber et al. (28) investigated if pre-therapeutic ^68^Ga-DOTATOC PET/MRI parameters were able to predict treatment response and evaluated which were the most significant changes that occurred after therapy both for conventional PET parameters and RFs. In contrast with our preliminary results, their study showed no statistically significant changes in PET parameters since neither PET nor ADC map parameters were predictors of therapy response. However, these data are not fully comparable with our results since different parameters and a different methodology have been applied. Moreover, the authors compared in their study the PET parameter changes between responders and non-responders in the entire cohort of 18 patients regardless of different treatments (PRRT and conventional therapies).

Regarding the two volumetric conventional PET parameters, the few data at present available in literature showed a significant correlation between SRETV_wb−50_ and disease progression. Tirosh et al. (25) observed an association between “^68^Ga-DOTATATE TV” (corresponding to SRETV_wb−50_) >7.0 ml with a higher risk for disease progression and “^68^Ga-DOTATATE TV” >35.8 ml which was associated with a higher disease-specific mortality. Toriihara et al. (26) showed an association between “^68^Ga-DOTATATE ∑SRETV” (corresponding to SRETV_wb−50_) >11.29 ml and shorter progression-free survival. In our study, the responder patient presented SRETV_wb−50_ of 35.2 ml at baseline PET/CT, just below the cutoff value associated with higher disease-specific mortality in the Tirosh study. The SRETV_wb−50_ in the non-responder patient was far above the cutoff reported earlier (493.8 ml). These data are consistent with the different response and outcome to PRRT of our patients, namely, considering the higher tumor burden and the relative lower uptake of the lesions in the non-responder vs. the responder patient (mean TSR 2.35 for patient A vs. 8.80 for patient B and mean TLR 7.44 for patient A vs. 24.81 for patient B). Overall survival after PRRT was 26 months (at last follow-up, the patient was alive with disease) in patient B, while it was 13 months in patient A (died with disease). On the other hand, the median absorbed dose received by the two patients was very similar; therefore, in these two cases, dosimetry cannot explain completely the different responses as well as negative pre-PRRT ^18^F-FDG-PET/CT (Figure 1). Furthermore, the opposite trend of TLSRE_wb−50_ and SRETV_wb−50_ (increase in patient A and decrease in patient B) in accordance with RECIST 1.1 might suggest a role for these parameters also in PRRT response. To our knowledge, there are no studies designed to evaluate the role of ΔSRETV_wb_ and ΔTLSRE_wb_ on therapy response.

### Limitation

This study is not exempt from limitations. The two patients were affected by different primary tumors. Moreover, patient A had liver metastases 1 year after the diagnosis and bone and nodal metastases 5 years after, while patient B had liver metastases 6 years after the diagnosis. Furthermore, the PRRT protocols were slightly different in the two patients due to the only recent approval of ^177^Lu DOTATATE. Several clinical parameters could alone predict different responses to PRRT and prognosis; nevertheless, in the future, volumetric PET parameters and radiomic features could be complementary.

Another limitation is represented by the sample size. However, this study was aimed to be a preliminary exploratory analysis to assess the feasibility of radiomic analysis applied in this clinical scenario. Thus, definitive conclusion cannot be drawn according to our data at this stage, and these promising results encouraged us to start a prospective study in NET patients eligible for PRRT to evaluate the application of RFs as predictors of therapy response.

All the variables affecting the robustness of RFs to improve reliability and reproducibility (such as segmentation methods, rescaling factor, and reconstruction algorithms) should be investigated in future studies. In our study, according to Bailly et al. (29), only GLRLM-RP and GLZLM-ZP could be considered as adequately robust over reconstruction algorithms.

## Conclusion

Despite having evaluated only two patients, this preliminary analysis suggests the use of RFs and TLSREwb-50 and SRETVwb-50 as parameters to evaluate response to PRRT in NET patients. Moreover, pre-therapy RFs and RF changes observed from pre- to post-therapy scan might help to predict and to assess response to PRRT, leading to optimization in the management of NET patients. These exploratory results need to be confirmed by future studies enrolling a larger and more homogenous population.

## Data Availability Statement

The original contributions generated for the study are included in the article/Supplementary Material, further inquiries can be directed to the corresponding author/s.

## Ethics Statement

All procedures performed in studies involving human participants were in accordance with ethical standards of the institutional and/or national committee and with the 1964 Helsinki declaration and its later amendments or comparable ethical standards. The use of the data of these two patients was approved by AOU Città della Salute e della Scienza di Torino Ethics Committee (IRB protocol: CS2/477), as part of a largest retrospective study.

## Informed Consent

Informed consent was obtained from all individual participants included in the study.

## Author Contributions

DD designed the study. VL reviewed the PET/CT scans. VL, OR, EG, and BDe collected the data and conducted the statistical analysis on PET/CT scans. FF and AV collected the data and conducted the statistical analysis on dosimetry. PT, FC, and BDi contributed in the review of the data and the statistical analysis. VL, OR, and DD wrote the manuscript. VL, OR, EG, FC, PT, LC, AP, FM, and DD discussed the results and commented on the manuscript. All the authors reviewed the final manuscript.

## Conflict of Interest

The authors declare that the research was conducted in the absence of any commercial or financial relationships that could be construed as a potential conflict of interest.
