# Supplementary material for: 68Ga-DOTATOC PET/CT-Based Radiomic Analysis and PRRT Outcome: A Preliminary Evaluation Based on an Exploratory Radiomic Analysis on Two Patients
Source: Front Med (Lausanne). 2021 Jan 26;7:601853. doi: 10.3389/fmed.2020.601853 (PMC7870479; doi:10.3389/fmed.2020.601853)
Supplement: Supplementary file 1 [file Data_Sheet_1.PDF]

SUPPLEMENTAL MATERIALS - TABLES

Table S1 – PET parameters (SUVmax and volume) of liver metastases, extracted from the manual segmented VOI (VOI_{ms}) of the pre-therapy and the pos-therapy PET/CT scan of both patients.

PATIENT A				
**LESIONS**	**^{68}Ga-DOTATOC PRE-PRRT**		**^{68}Ga-DOTATOC POST-PRRT**	
	**SUVmax**	**VOLUME (voxel)**	**SUVmax**	**VOLUME (voxel)**
**Liver 1**	17.7	7042.0	17.8	13572.0
**Liver 2**	12.9	1104.0	8.2	2546.0
**Liver 3**	17.7	4208.0	17.8	7066.0
**Liver 4**	10.0	2740.0	12.8	6773.0
**Liver 5**	8.1	1010.0	12.1	3798.0
**Liver 6**	14.1	292.0	17.1	366.0
**Liver 7**	35.9	2844.0	27.1	2184.0
**Liver 8**	10.4	109.0	19.8	169.0
**Median (min – max)**	13.5 (8.1 – 35.9)	1922.0 (109.0 – 7042.0)	17.4 (8.2 – 27.1)	3172.0 (169.0 – 13572.0)

PATIENT B				
**LESIONS**	**^{68}Ga-DOTATOC PRE-PRRT**		**^{68}Ga-DOTATOC POST-PRRT**	
	**SUVmax**	**VOLUME (voxel)**	**SUVmax**	**VOLUME (voxel)**
**Liver 1**	93.2	1274.0	63.7	480.0
**Liver 2**	13.9	62.0	5.1	53.0
**Liver 3**	31.0	157.0	6.9	63.0
**Liver 4**	14.7	109.0	4.0	41.0
**Liver 5**	22.5	160.0	5.7	40.0
**Liver 6**	104.4	691.0	3.9	77.0
**Liver 7**	85.5	385.0	5.0	74.0
**Liver 8**	17.2	90.0	2.3	23.0
**Liver 9**	76.2	590.0	6.9	111.0
**Liver 10**	20.3	110.0	4.2	50.0
**Median (min – max)**	26.7 (13.9 – 104.4)	158.5 (62.0 – 1274.0)	5.1 (2.3 – 63.7)	58.0 (23.0 – 480.0)

Table S2 – *P*-values of Mann-Whitney U-test comparing the radiomic features value extracted by the liver lesions VOI_{ms} of the pre-PRRT PET/CT of the two patients.

RADIOMIC FEATURES	p-value	RADIOMIC FEATURES	p-value
CONVENTIONAL-SUVmin	0.536	**GLRLM_LRE**	0.001
CONVENTIONAL-SUVmean	0.230	GLRLM_LGRE	0.351
**CONVENTIONAL-SUVstd**	0.025	GLRLM_HGZE	0.071
**CONVENTIONAL-SUVmax**	0.036	GLRLM_SRLGE	0.174
CONVENTIONAL-SUVpeak 1mL	0.222	**GLRLM_SRHGE**	0.042
**CONVENTIONAL-TLSRE (mL)**	0.025	GLRLM_LRLGE	0.408
**HISTO_Skewness**	0.001	GLRLM_LRHGE	0.918
**HISTO_Kurtosis**	0.007	**GLRLM_GLNU**	0.001
**HISTO_ExcessKurtosis**	0.007	**GLRLM_RLNU**	0.023
HISTO_Entropy_log10	0.230	**GLRLM_RP**	0.002
HISTO_Entropy_log2	0.230	NGLDM_Coarseness	0.142
HISTO_Energy (uniformity)	0.417	**NGLDM_Contrast**	0.001
**SHAPE_Volume (mL)**	0.003	**NGLDM_Busyness**	0.001
**SHAPE_Volume (voxel)**	0.003	**GLZLM_SZE**	0.001
SHAPE_Sphericity	0.070	**GLZLM_LZE**	0.001
**SHAPE_Compacity**	0.003	GLZLM_LGZE	0.174
**GLCM_Homogeneity**	0.002	**GLZLM_HGZE**	0.008
GLCM_Energy	0.210	GLZLM_SZLGE	0.252
**GLCM_Contrast**	0.001	**GLZLM_SZHGE**	0.003
**GLCM_Correlation**	0.008	**GLZLM_LZLGE**	0.002
GLCM_Entropy_log10	0.091	**GLZLM_LZHGE**	0.003
GLCM_Entropy_log2	0.091	GLZLM_GLNU	0.210
**GLCM_Dissimilarity**	0.001	GLZLM_ZLNU	0.174
**GLRLM_SRE**	0.001	**GLZLM_ZP**	0.001
